# Towards 3D characterisation of site-controlled InGaAs pyramidal QDs at the nanoscale

**DOI:** 10.1007/s10853-022-07654-2

**Published:** 2022-08-30

**Authors:** Kristina M. Holsgrove, Tamsin I. O’Reilly, Simone Varo, Agnieszka Gocalinska, Gediminas Juska, Demie M. Kepaptsoglou, Emanuele Pelucchi, Miryam Arredondo

**Affiliations:** 1 School of Mathematics and Physics, Queen’s University, Belfast, UK; 2 University of Glasgow, Glasgow, G12 8QQ UK; 3 Tyndall National Institute, “Lee Maltings”, University College Cork, Cork, Ireland; 4 SuperSTEM Laboratory, SciTech Daresbury Campus, Daresbury, WA4 4AD UK; 5 Department of Physics, University of York, York, YO10 5DD UK

## Abstract

In this work, we report an extensive investigation via transmission electron microscopy (TEM) techniques of InGaAs/GaAs pyramidal quantum dots (PQDs), a unique site-controlled family of quantum emitters that have proven to be excellent sources of single and entangled photons. The most striking features of this system, originating from their peculiar fabrication process, include their inherently 3-dimensional nature and their interconnection to a series of nanostructures that are formed alongside them, such as quantum wells and quantum wires. We present structural and chemical data from cross-sectional and plan view samples of both single and stacked PQDs structures. Our findings identify (i) the shape of the dot, being hexagonal and not triangular as previously assumed, (ii) the chemical distribution at the facets and QD area, displaying clear Indium diffusion, and (iii) a near absence of Aluminium (from the AlAs marker) at the bottom of the growth profile. Our results shed light on previously unreported structural and chemical features of PQDs, which is of extreme relevance for further development of this family of quantum emitters.

**Graphical abstract:**

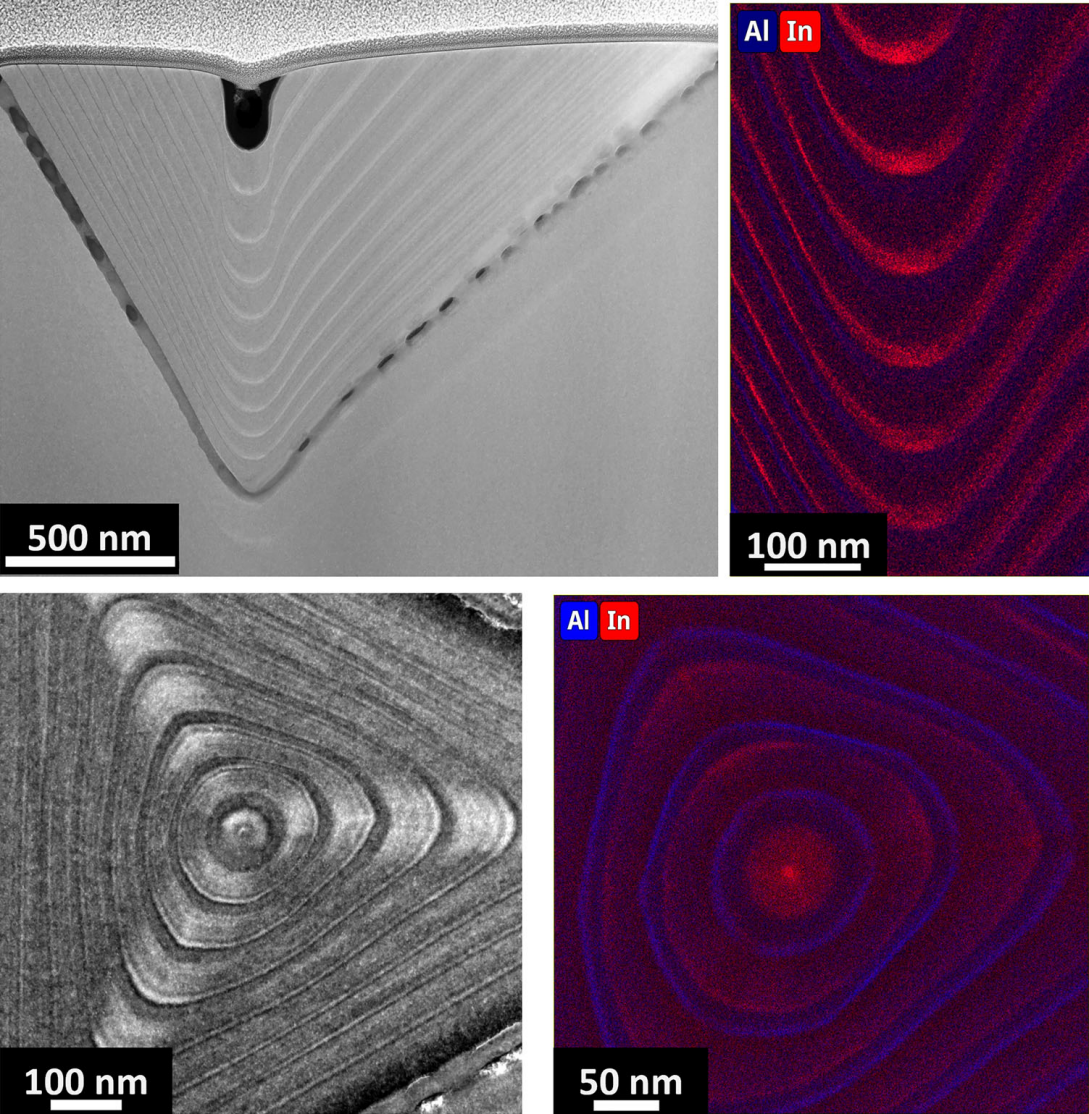

**Supplementary Information:**

The online version contains supplementary material available at 10.1007/s10853-022-07654-2.

## Introduction

Metal–organic vapor phase epitaxy (MOVPE) has been one of the workhorses of semiconductor fabrication since it was first developed [1], alongside molecular beam epitaxy (MBE) and other deposition techniques [2]. However, in all of these techniques, epitaxial growth is mostly performed on planar substrates with a specific and uniform crystal orientation, as the use of a more complex topography is usually detrimental to the quality of the growth itself and significantly complicates the processing of samples afterwards. Examples of growth of 3-dimensional structures, made via vapor–liquid–solid epitaxy are well reported in the literature [3], but even these often start from a planar substrate and can be considered as a different topic altogether. In the 1980s, interest emerged in the growth of epitaxial layers on 3-dimensional (3D) templates in the form of V grooves via MOVPE: these were the first examples of III–V self-assembled low-dimensional structures ever published, pre-empting the era of quantum dot research which emerged a few years later [4, 5]. The growth of epitaxial layers on 3D templates was initially motivated by the new possibilities this approach held for the fabrication of efficient lasers [6, 7] while at a later stage interest greatly increased in using this growth strategy to address more fundamental aspects of semiconductor physics [8, 9]. For example, in the V-groove system, it was first observed that the non-planar nature of the template plays a key role in the mechanisms of MOVPE growth, determining a growth profile and alloy stoichiometry that is very different from a uniform growth on all the exposed surfaces, as one would be tempted to assume. Years later, it became clear that the growth on 3D templates is dictated by the competition of the decomposition kinetics of the metal–organic precursors [10], which is crystal-plane dependent and involves adatom diffusion and sticking mechanisms [5]. The former effect results in the preferential deposition and growth on specific crystallographic planes, while the latter causes an evolution of the growth profile which tends to develop a flat base at the bottom of the groove (generally referred to in the literature as a self-limited profile). Additionally, the different adatom diffusion coefficients can introduce a spatial dependence of material stoichiometry during the growth of ternary alloys [11–15].

**Figure 1:**
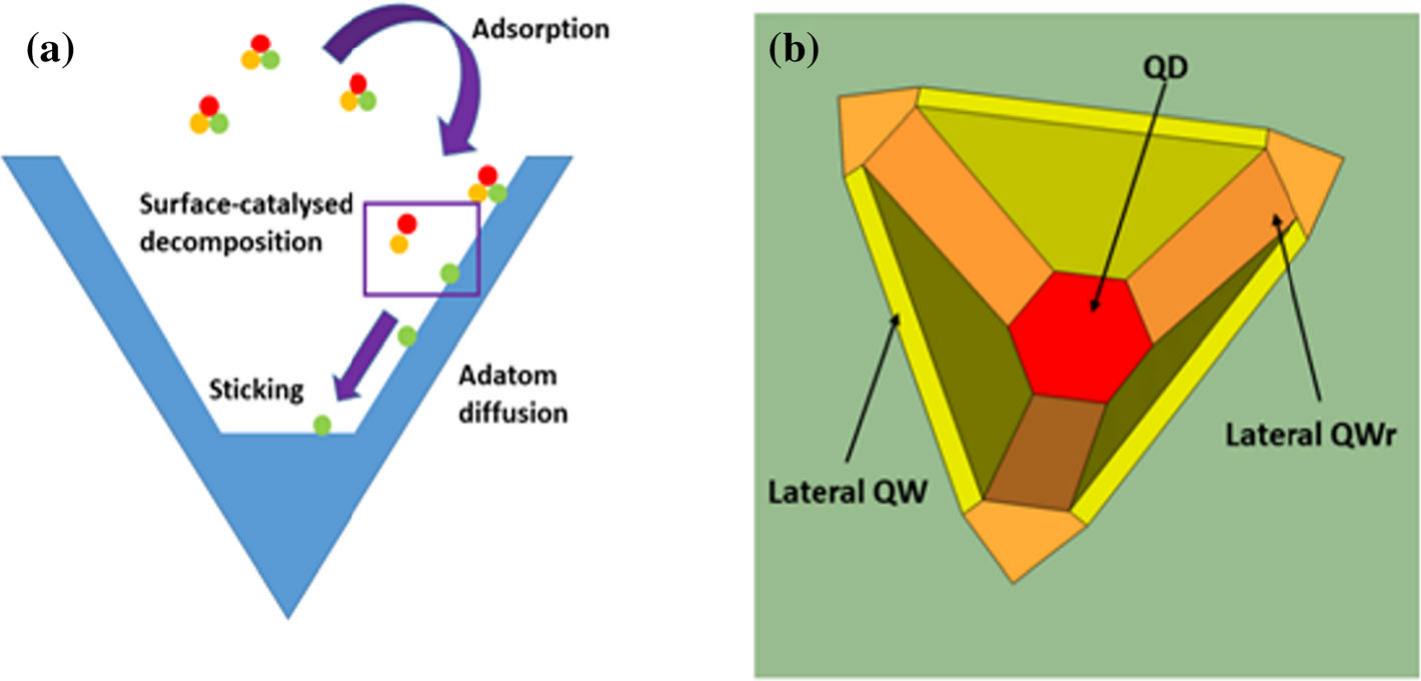
Sketch of **a** the MOVPE process on V-grooves and **b** typical nanostructures formed growing InGaAs between GaAs layers in a tetrahedral recess. The sketch indicates the InGaAs structures (having different In concentrations: yellow, orange and red), which are then cladded by the GaAs layers (not shown here)

Even more interesting effects emerge when instead of V-grooves, alternative 3D templates are considered^1^. It is possible to perform MOVPE growth on inverted tetrahedral recesses defined on 111B-oriented GaAs wafers, where the peculiar growth mechanisms allow, in principle, for the fabrication of quantum emitters along the axis of the tetrahedron if a very thin layer of semiconductor material is confined by barriers possessing a higher bandgap. This family of nanostructures, called pyramidal quantum dots (PQDs) due to the shape of the growth template being an inverted tetrahedral pyramid, have proven to be excellent sources of single and entangled photons [16–19] upon optical excitation or electrical charge injection, and are particularly attractive due to their site-controlled nature, as they are deterministically formed along the axis of a lithographically defined inverted tetrahedron (see Fig. 1): they can be considered more suitable candidates for integration in scalable quantum technologies when compared to self-assembled quantum dots (QDs), which tend to grow at random locations on a wafer. Of note, interesting applications embedding PQDs into a PIN-junction device have been previously demonstrated as valuable sources of quantum light [20, 21]. Moreover, this fabrication strategy has proven to be a suitable way to stack multiple dots to create more complex, molecule-like structures [22, 23].

PQDs are indeed unique in the quantum technology landscape, and despite a large number of results demonstrated to date for this QD system, and its significant potential for quantum technologies, there are surprisingly very few reports [24, 25] providing a detailed morphological and chemical characterisation at the micro and nanoscale, whereas its historic and conceptual ancestor, the V-groove quantum wire system, has been extensively characterised [14, 26]. Alternatively, theoretical models have been developed to describe how the various growth parameters should determine the shape and composition of the dots in these PQDs [4, 27], however, the lack of experimental validation of the latter via direct assessment of the morphology and composition of the dots and surrounding structures hampers further development in the field. Even the exact shape of the dot, and thus of the confining potential is still unknown: considerations of the shape of the growth template suggest that it should belong to the C_3v_ symmetry group. This has so far been the starting point of all ab initio simulations of PQDs [28–31] along with the approximation of a rather uniform composition of the dot itself. The work here presented proves that both assumptions are incorrect and pave the way for accurate ab initio simulations of the excitonic states of this family of quantum dots.

The exact shape and symmetry of the dot are of great importance, as it is one of the main features that determine the magnitude of the so-called excitonic fine-structure splitting (i.e. the lifting of energy degeneracy between the two bright excitonic states, which is induced by structural asymmetries), and in turn the capability of the dot to emit entangled photons [32]. In general, an accurate understanding of the exact geometry would be critical not only for a more comprehensive electronic state description but also for testing more advanced quantum technology proposals, for example, the predictions of novel entangled photon emission schemes based on “quantum dumbbells” [33] and light-hole/heavy-hole mixing effects.

To complicate the matter even further, the QD is usually formed alongside, and is interconnected to, a series of additional nanostructures, which are formed due to the different diffusion coefficients of the group III adatoms such as Indium and Aluminium; if an InGaAs QD is grown between GaAs barriers, it will be connected to three lateral InGaAs quantum wires (QWRs) and three lateral quantum wells (QWs), as shown in Fig. 1b, whereas if a GaAs dot is grown between AlGaAs barriers, it will be surrounded by two vertical QWRs grown along the axis and three vertical quantum wells. Far from being a nuisance, these features could be exploited to selectively inject charges in the QDs in electrical pumping schemes [20], but they also create a cluttered environment in the surroundings of the QDs. Thus, extreme caution must be exercised both in sample preparation and in data analysis to localise the specific nanostructure of interest.

Certainly, the system in question is extremely challenging to investigate using microscopy techniques. As discussed before, the MOVPE growth mechanism and reactor environment couple to the tetrahedral template to determine a fully 3-dimensional outcome, instead of the 2-dimensional one with translational symmetry obtained starting from V-grooves. All this makes experimental methods routinely used to directly inspect epitaxial layers such as high-energy electron diffraction (RHEED), atomic force microscopy (AFM) or even X-ray diffraction (XRD) non-applicable for this system, and reports with more detailed data using high-resolution techniques such as transmission electron microscopy (TEM) are very limited [24, 25].

Thus, the aim of this work is to investigate this fascinating and unique, yet challenging, family of site-controlled quantum emitters. We report, for the first time to our knowledge, on the structural and chemical nature of InGaAs/GaAs PQDs grown in large pitch recesses by TEM techniques. To better map the shape and chemical composition of the QD, we have analysed both plan view and cross-sectional samples that yield complementary information.

## Materials and methods

***Epitaxy***: Sample preparation starts by defining arrays of inverted tetrahedral holes on a (111)B-oriented semi-insulating GaAs wafer using optical lithography and wet etching in a Br:MeOH solution, where the anisotropy of the etching is exploited to expose the three (111)A facets of the inverted tetrahedron. Epitaxial growth is then performed at a thermocouple temperature of 730 °C in an Aixtron MOVPE reactor using (industry standard) trimethylgallium, trimethylindium, trimethylaluminum and high purity arsine as precursors, with (> 9 N) purified nitrogen as a carrier gas. All quoted thickness when discussing sample growth should be intended as nominal values. The interplay of the different decomposition kinetics of the precursors on the various crystal planes and adatom diffusion processes allows for growth to take place almost exclusively on the (111)A surfaces and a single QD (or multiple stacked ones) can be deterministically fabricated along the axis of the tetrahedron [18, 23] if a thin layer of semiconductor material is grown between two layers having a higher bandgap (see Fig. 1).

***Sample design***: The manuscript presents the analysis of one PDQ system in two different structures:

A) *A single QD pillar structure:* A 3 nm ${\mathrm{In}}_{0.25}\mathrm{GaAs}$ dot was grown between two 30 nm thick GaAs barriers. Thin (20 nm) ${\mathrm{Al}}_{0.96}\mathrm{GaAs}$ layers were grown before and after the barriers to provide markers that could assist in the location of the dot during TEM sample preparation, due to the contrast displayed between the Al-rich layer and the rest of the epitaxial material (see full details in Table S1). The markers are found on both the QW and QWR sides.

In order to improve the odds of correctly locating the dots, some PQD samples were further processed to obtain pillars using a top-down process [34]. This was achieved by depositing a 300 nm thick SiO_2_ hard mask via sputtering and, by exploiting the 3-dimensionality of the system, exposing the GaAs using chemo-mechanical planarization (CMP) [35], while leaving some SiO_2_ on top of the structure, as shown in Fig. S1. This process ensures the fabrication of a dielectric hard mask which is perfectly aligned to the epitaxial structure underneath and to the dot contained therein (along the central axis), while the lateral size of the mask can be precisely controlled by adjusting the amount of material removed by CMP. Finally, pillars are fabricated via dry etching in an Oxford Cobra 100 plasma etcher, using an optimized BCl_3_/Cl_2_/Ar/N_2_ recipe (13 sccm BCl_3_, 13 sccm Cl_2_, 13 sccm Ar, 6 sccm N_2_, RF platen power 60 W, ICP power 600 W, pressure 4 mTorr) that ensures vertical and smooth sidewalls. This strategy allows for a more precise cut near the QD with a higher reproducibility, as the position of the QD can be clearly identified from the vertices of the SiO_2_ mask that act as alignment marks, and the lateral size of the system and of the recess can be shrunk from microns to hundreds of nanometers. In addition, the AlAs markers aid with the identification of the QD position.

B) *A multiple, stacked, QDs structure:* A sample was grown with QDs stacked along the axis of the tetrahedron. AlAs markers were introduced to facilitate the imaging of each period of the repeated structure. These also allowed to provide a rough estimation of the depth of the various dots in cross-sectional samples. The structure of the sample is detailed in Table S2. It was also decided to lower the nominal thickness of the dot from 3 to 2 nm (compared to A, the single QD structure) to reduce the risk of strain-induced relaxation in the stacked structure due to the InGaAs/GaAs lattice mismatch.

**Figure 2:**
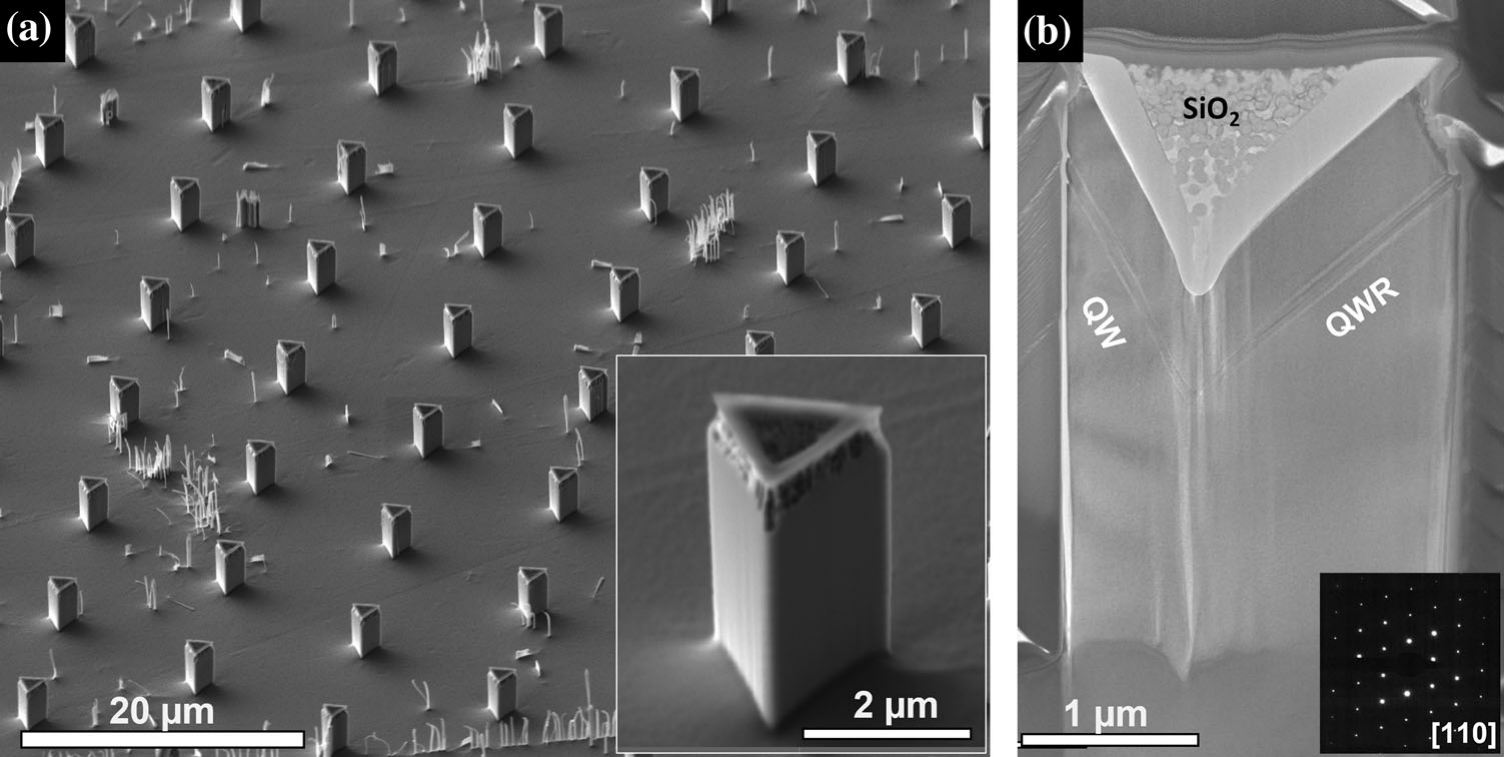
Overview of the nanopillars PQD structure. **a** Tilted secondary electron SEM image and **b** bright field TEM along [110] indicating the lateral quantum wire (QWR) and lateral quantum well (QW), where the brighter, lines are the AlGaAs markers. The selective area diffraction pattern is shown as an inset in (**b**)

***TEM Sample preparation***: Growth models and previous characterizations suggest that PQDs are much thinner along the growth direction. Thus, TEM analyses on cross-section and plan-view lamellae were performed. The more conventional cross-section samples are expected to give a more precise chemical information near (or at) the dot due to smaller averaging effects (as the dot-thickness is expected to be closer to the thickness of the lamella), while the plan-view samples would allow identification of the structure’s geometry more accurately, e.g., the shape of the dot in the plane perpendicular to the growth direction. This is of paramount importance, as previously discussed, to address the parameters affecting the magnitude of the excitonic fine-structure splitting [32]. A Lyra3 TESCAN dual beam scanning electron microscope was used to acquire secondary electron images, and FIB was used to prepare cross-sectional and plan view (PV) TEM samples which were further milled using an Ar^+^ beam in a PIPS II. For the PV samples, e-beam and ion-beam Pt was sputtered on the area of interest, followed by trench milling (1 nA), left cut (500 pA) and undercut (300 pA). The sample was lifted out in situ using the in-built nanomanipulator system and then rotated through 90° using a second needle attached to the stage, a series of attach and detach steps and two-stage rotations of 45°. Following this, the sample was attached to a Cu grid, and ion-beam Pt was sputtered (~ 2.5 μm, 100 pA) to protect the new top surface. The sample was then thinned on the grid until the pyramid was exposed, before a final 5 kV polish took the sample < 100 nm. See Figs. S2 and S3. Site-specific TEM sample preparation by FIB, in its many variations, is a well-established technique [36, 37]. However, in 3D structures like PQDs, there is still the challenge associated with accurately locating the QD. In general, while FIB allows for a more accurate cut at the centre of the pyramid, there is always some uncertainty in the final position, due to the lamella thickness and curtaining effects. The latter is mainly caused by the intrinsic pyramidal shape but also due to the presence of the residual SiO_2_ mask, as observed in Fig. 2b. Moreover, factors like small tilts in the final milling stages -removal of tens of nms- can affect the characterisation and induce a variation in the lamellae thickness, missing the exact position of the QD.

***TEM analysis:*** Bright field TEM and high-angle annular dark-field (HAADF) scanning TEM (STEM) images were acquired on a Thermofisher Talos F200X fitted with a Super-X energy-dispersive X-ray spectrometer (EDX) operated at 200 kV. Further STEM HAADF micrographs for geometrical phase analysis (GPA) and strain measurements were acquired on a Nion UltraSTEM 100MC, operated at 60 kV. The inner – outer collection angles for the HAADF STEM imaging were 90 and 200 mrad, respectively.

## Results and discussion

### Cross-sectional analysis of PQDs

An overview of the pillar structures containing a single PQD is shown in Fig. 2a. A highly ordered array with symmetric and uniform pyramidal structures can be observed, while the inset shows a closer look at a representative PQD pillar. The average dimensions of these pillars are ~ 3 µm height with a triangular base 1.5 µm long. The typical cross-section structure for MOVPE grown PQDs, an inverted pyramidal pattern with a QD at the apex of the pyramid (at the bottom of the inverted recess), is clearly observed in the representative bright field TEM cross-sectional micrograph in Fig. 2b. The central ‘drop’ shape has been previously reported as a characteristic feature of the tip [27], indicating the centre of the pyramid.

**Figure 3:**
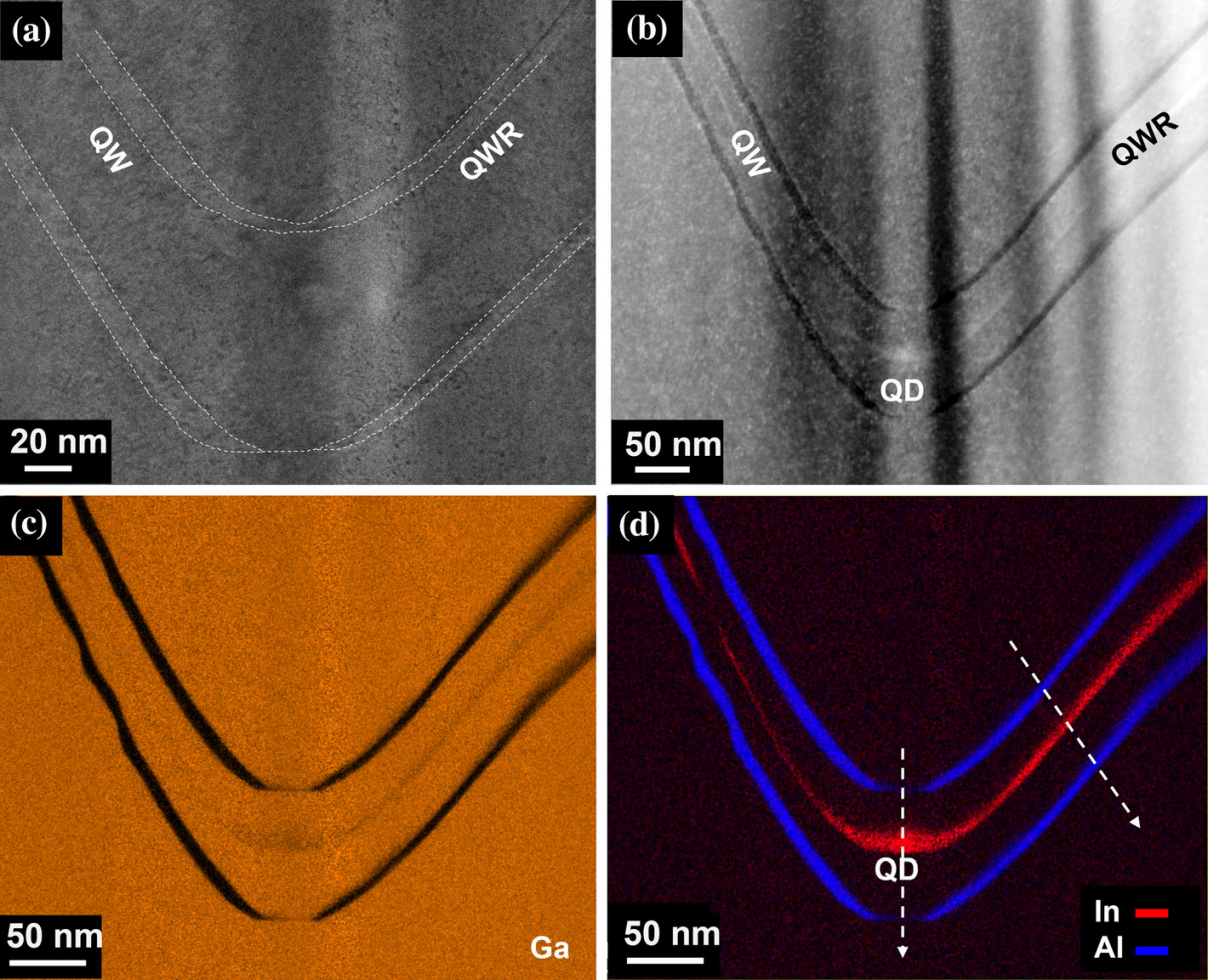
Cross-section of the PQD structure containing a single QD. **a** TEM overview of apex of the pyramid (at the bottom of the inverted recess) indicating the Al markers by white dotted lines, **b** HAADF STEM overview and EDX elemental maps for **c** Ga, and **d** In and Al, red and blue respectively. The white dotted lines are representative of the positions from which the line profiles for Fig. 4 were acquired from

Depending on the imaging conditions, the contrast variation from TEM micrographs can be used to provide quantitative information about strain and composition of the sample. Conventional bright-field TEM mode is not enough to distinguish the thin, 3 nm, ${\mathrm{In}}_{0.25}\mathrm{GaAs}$ QD (Figs. 2b and 3a) however, the thicker AlGaAs markers are clearly visible; the markers thickness in the lateral quantum well (QW) side range between ~ 11 and 13 nm thick and ~ 5–6 nm in the lateral quantum wire (QWR) side. While small differences between the nominal and measured thickness are routinely observed, the more drastic difference between the sides of the pyramid can also be attributed to small projection effects. The ${\mathrm{In}}_{0.25}\mathrm{GaAs}$ layer in the (111)A facets can be more easily identified from STEM HAADF where a higher contrast between GaAs and InGaAs layers is achieved due to the Z-contrast condition. Thus, HAADF is the imaging mode used for analysing all samples here presented.

The overall growth profile shows a pronounced dip in the centre with a drop shape. Depending on the cut position of the lamella with respect to the pyramid tip, a change in the vicinal facet angle with respect to the growth direction can be observed. It was previously reported that the GaAs facet evolving during growth on the sidewalls of the pyramidal hole is not the original (111)A, but a vicinal one, with the angle between the vicinal (111)A and the base facet (111)B reported to be ~ 77° [27]. Direct measurements on Figs. 3a and 3b show an angle variation of the vicinal facets of ~ 64° for the QW side and ~ 55° for the QWR side. These angles decrease toward the tip with values as low as ~ 18° for the QW side. This highlights a rather complex evolution of the angle between the base of the growth profile and the sidewalls of the pyramid.

Pseudomorphic epitaxy of ternary alloys, coupled with the mechanisms determining MOVPE growth on 3D patterned substrates, might cause a rather complex distribution of strain within the epitaxial structure which, as previously mentioned, mainly stems from the spatial variations of the alloys’ stoichiometry as well as from the faceting. As a complementary study, we investigated the strain and chemical distribution across the structure. For the strain, geometrical phase analysis (GPA) was carried out across both the InGaAs-GaAs and AlGaAs-GaAs layers on the sides of the pyramid (see Fig. S4), both confirm very low strain fields (< 0.1%). In general, any possible Indium segregation originates due to adatom diffusion effects, and while strain could indeed have a role influencing the diffusion during growth, in these particular structures strain seems to play a minor role, at least at the sides of the pyramid. This has been previously addressed by models that accurately describe the In segregation and model the corresponding emission [27].

We now turn our attention to the chemical composition. Figure 3b–d display representative HAADF images and corresponding EDX elemental maps for Al-Kα, Ga-Kα and In-Lα lines from the sample shown in Fig. 2 (Arsenic is not shown here, as the concentration is uniform in the epitaxial material). Before discussing the composition near the QD area, we will first discuss the appearance of the AlAs markers. While the vicinal (111)A planes clearly display the AlAs layers as expected, a striking feature is the flattening and the near absence of Aluminium at the bottom of the growth profile, which is found in all cases where the lamella is cut at the centre of the pyramid. Although the effect was somehow expected, its magnitude is quite surprising. If GaAs is grown, a large (~ 60 to 80 nm wide) (111)B base is expected at typical growth temperatures, whereas the lower diffusion coefficient of Al adatoms tends to produce a much smaller self-limited profile at the bottom (~ 10 nm). This is confirmed by growth models [27, 38–40] which predict a different self-limited profile (i.e., lateral extension at the bottom) for different alloys.^1^ Transitioning from one material to the other implies that growth is performed in non-equilibrium conditions, resulting in a transient that is resolved when the self-limited profile has evolved to reach one of the alloys being grown. In this case, the thickness of the AlAs layer was too small to resolve the transient and resulted in a layer which is both much thinner and nonuniform due to the spreading of Al adatoms over a surface whose area was dictated by the underlying GaAs layer, and much larger than the ideal AlAs self-limited profile.

Interestingly, our analysis provides valuable insight into what happens when the growth is performed far away from equilibrium conditions and investigates, for the first time, the beginning of the transient. With some surprise for the authors, these results indicate that AlAs avoids, in the early stages of the deposition, to follow the rule requiring a common vertical growth rate (even when considering corrections for the expected differences necessary to bridge from one self-limited profile to another), with the sidewalls growth rate largely decoupled from the centre one (there is no obvious gradual transient from the sidewalls to the (111)B surface). The extremely thin AlAs layer in the centre implies fundamentally a different recess geometry organization than the one originally imposed by GaAs, and presumably the formation of extra faceting. The subsequent GaAs growth on the other hand seems to be re-establishing the original equilibrium in less than a few tens of nms, also an unexpected result. This finding is of extreme interest for engineering PQD structures. It implies that it should be possible, in principle, to engineer QD structures with a rather extreme high band gap lateral confinement by growing relatively thin AlAs layers directly on GaAs, while the vertical confinement would be significantly less accentuated, if not vanishing. This would present an alternative route to selective carrier injection to the one exploited by Chung et al. [20]. And, also of particular interest for the engineering of GaAs/AlGaAs, especially GaAs/AlAs PQDs, which have not been investigated in this work but have already been reported in the literature [17], where, the thin AlAs barrier in the latter case might be the reason for the unexpectedly high values of fine structure splitting reported, due to a leak-out of the wavefunctions.

Assessing the features near the QD, it can be inferred from Fig. 3d that some Indium segregates at the apex of the pyramid (at the bottom of the inverted recess). The highest Indium concentration is found at the centre (tip of the pyramid) where it appears as a rather diffused ~ 20 nm thick layer. The dot lateral spatial broadening is similar to that reported in core–shell nanowires [41]. This broadening could be attributed to two well-known effects, electron beam broadening and electron channelling [42–44]. However, the beam broadening would show a symmetrical effect throughout the structure, which is not the case and electron channelling should not be a dominant effect for an uncorrected TEM, for the convergence angle used here. Moreover, it has been shown that the [110] direction of InGaAs is better suited for HAADF-STEM quantification [45]. Thus, the Indium diffusion (and broadening of the dot) here observed is considered as a real effect, a direct result of the growth conditions. Previous reports on planar structures have showed that, for example, the growth temperature plays a key role in the In distribution and interface roughness of (GaIn)As QWs [46], although it is unknown if the growth temperature would have similar effects in these PQDs. It should be mentioned that it is known that most standard EDX quantification methods have some degree of uncertainty, thus the atomic fractions here reported should be taken with some caution. That said, it should be noted that the higher contrast observed in HAADF imaging supports the evidence of Indium diffusion. The fact that the Indium content is highest at the bottom of the tetrahedral structure is not surprising in view of the diffusion length of Indium being higher than that of Ga. This has been previously reported in V-groove quantum wires [14, 47], but theoretical modelling on PQDs previously published based on comparison of photoluminescence data, suggested a significantly smaller effect in QDs, while maintaining a relevant effect in the lateral wires [23]. The In distribution is of relevance as it provides further information on the effective scale of group IIIA atoms migration to the bottom of the (111)B planes while confirming that the diffusion length of Indium is much higher than that of Ga for all of the facets here present [27, 48].

**Figure 4:**
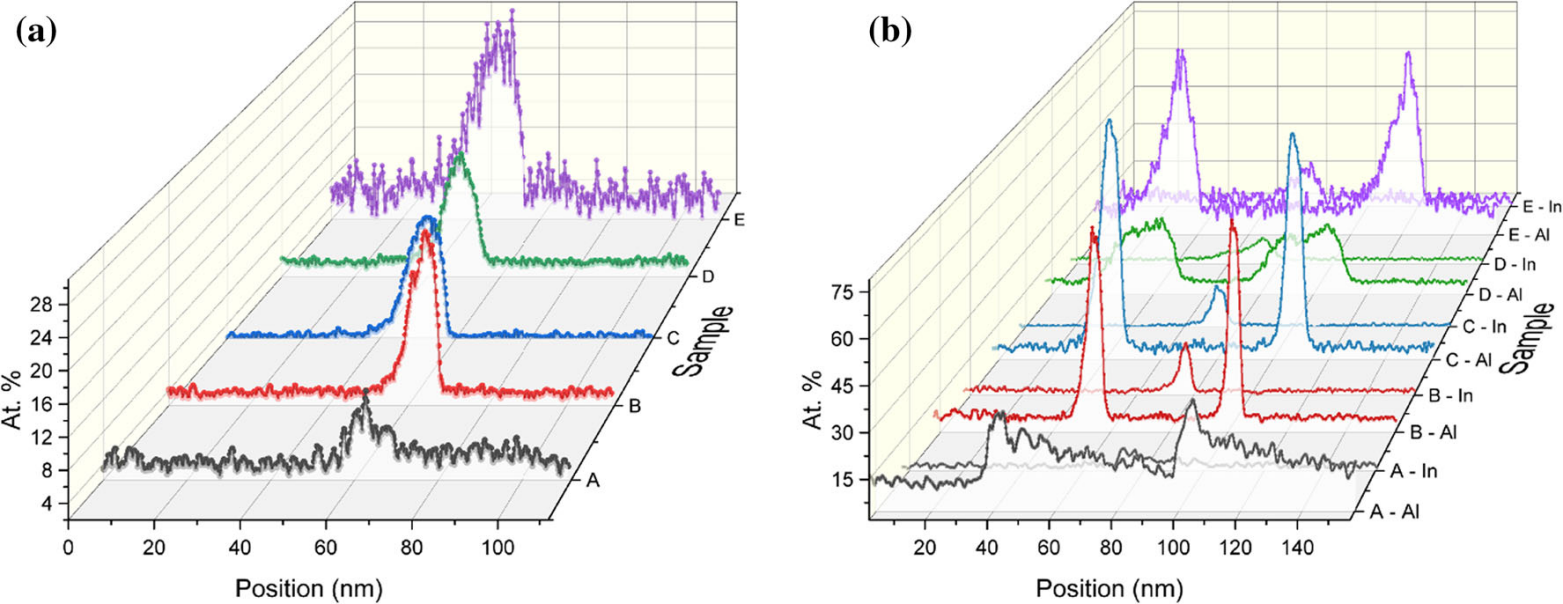
Atomic fractions profiles for five different cross-sectional samples, A to E, of single PQDs from the same bulk sample, extracted from the representative positions marked in Fig. 3d by the white dotted lines. **a** Indium content near the pyramid tip and **b** Indium and Aluminium content at the quantum wire (QWR) side. For each sample, the Al content is given first (bottom) and the In content follows (top)

Looking at the wire and well structures, the Indium concentration varies throughout the structure, shown by a gradual change in composition along the sides of the pyramid, and it can also be observed that the composition change from one facet to the other is not abrupt. We have examined several cross-sectional lamellae from the single PDQ structure and observed the same general trend, with the chemical composition changing as a function of position (FIB milling cut). The measured concentration values for both Indium and Aluminium vary, depending on how aligned the cross-section samples are with respect to the apex of the pyramid (at the bottom of the inverted recess). Figure 4 displays the atomic fraction concentration for several lamellae from single PQDs within the same bulk sample examined in this work. The maximum concentration of Indium is always located at the tip of the pyramid, where the QD is located, with an average Indium (In) concentration between 26 and 12 at.% (Fig. 4a). It should be noted that at this point the Aluminium concentration is at its lowest (as seen in Fig. 3d). Similarly, the concentration of Aluminium and Indium differs drastically for the QWR and the QW (the pyramid sides). The Indium concentration is at its highest on the QWR side and in contrast, the Aluminium concentration is at its lowest, see Fig. 4b. On the QW side, the Al concentration is lower and fluctuates across the structure as a function of distance. In general, the Al concentration is less diffuse on the QW side; similarly, the In concentration is lowest on the QW side in comparison to the QWR side (see Fig. S5). Undoubtedly, the small difference in the position of the FIB milling and potential associated projections, play a crucial role when examining these 3D structures. The full EDX elemental maps for the samples from Fig. 4 can be seen in Fig. S6.

### Plan-view analysis of PQDs

**Figure 5:**
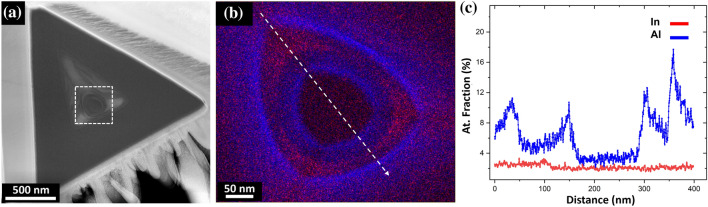
Plan view of the pillar PQD system containing a single QD. **a** Overview HAADF STEM image, **b** EDX elemental map from the area marked by a white dotted box in (**a**), for In (red) and Al (blue), and **c** atomic fraction profile from the area marked by the white arrow in (**b**)

While the traditional cross-sectional analysis of PQDs provides significant information about their structure and composition, it is far from being complete as it does not provide any information on their actual in-plane shape. Figure 5 displays one of the attempts to capture the single QD from Fig. 4b in plan-view. It can be seen that the cut is not perfectly aligned, indicated by the skewed shape of the central feature and the non-equidistant lengths from the wires. This is more clearly seen by the lack of a central In-rich feature in the EDX analysis (Fig. 5b and c).

**Figure 6:**
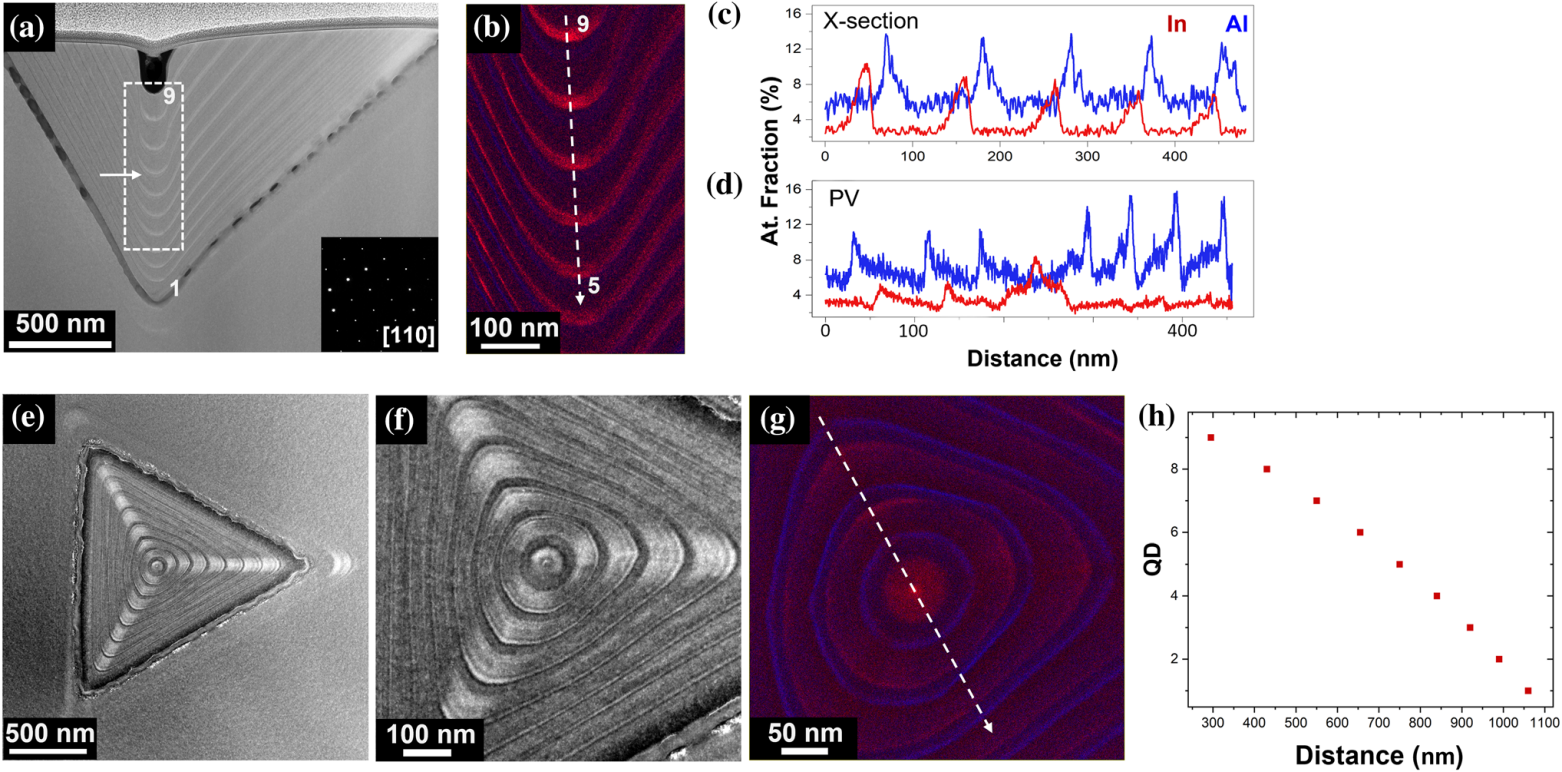
Stacked QDs PQD structure. **a** HAADF STEM overview image indicating the number of QDs: 1–9, **b** Al and In EDX elemental maps from the section marked by the white square rectangle in (**a**), **c** and **d** show the Al and In atomic fraction for QDs 9 to 5 (cross-section in (**b**)) and across the plan view, PV, (as marked in (**g**)), respectively. **e** and **f** are HAADF STEM overviews of the plan view sample, roughly acquired from QD4 as marked by the white arrow in (**a**). **g** is the corresponding Al and In EDX elemental map and **h** shows the spacing of the stacked QDs in the cross-section sample. In all cases Al is blue and In is red

With the intention to analyse another structure which is also of great interest [23], while increasing the probability of cutting through a QD, it was decided to use a sample with multiple QDs structure of the same InGaAs PQD system. In this case, the stacked QDs are separated by AlAs markers. Figure 6a shows the HAADF overview image of the cross-sectional sample, where 9 QDs can be observed, once more the shape of the drop indicates the position of the cut closely aligned to the apex of the pyramid. Similar to the single PQD system, EDX reveals that the QDs are also diffuse, with the In spreading ~ 25 nm in height. Additionally, the average In concentration is at its highest for the last grown layers of the pyramid, at the QD marked as 9 in Fig. 6a, and this concentration progressively decreases towards the QDs near the apex of the pyramid (Fig. 6b and c). This variation (a progressive decrease) in In concentration for stacked QDs (from 9 to 1) is somehow not surprising, given that the growth (which proceeds from the apex of the pyramid and fills the recess) is known to evolve progressively with steeper vicinal (111)A as the recess is filled. Local effective growth rates change also (see below discussion). It is likely that this has an effect on the resulting self-limited profile and segregation, even if no modelling exists to date describing in detail these dynamics.

As it can be observed from the HAADF image in Fig. 6a, the spacing of the QD is not homogeneous and the QDs are closer together at the top of the pyramid with a separation of ~ 70 nm for the first 3 QDs and as much as ~ 115 nm for the last QDs at the bottom of the PQD structure (Fig. 6h). This is a direct result of constant nominal growth parameters: during MOVPE growth, the continuous filling of the recesses reduces the opening total surface. As all material deposited on the planar areas is known to diffuse towards the recesses and incorporate therein, the effective growth rate at constant nominal deposition rate is known to increase with growth time [10, 49], as the effective growth surface is reduced with growth time.

Figure 6e shows a representative overview of a plan view sample obtained from ~ 600 nm from the top of the pyramid, position marked by the white arrow in Fig. 6a. A general triangular shape can be clearly seen, the centre is positioned ~ 875 nm away from the corners, and the three lines of brighter contrast come from the lateral wires. Surprisingly instead, the QD displays an almost hexagonal geometry, better seen in Fig. 6f. This is interesting because so far, the exact self-limited profile of the dot had been unknown, but generally accepted that it would possess C_3v_ symmetry and mimic that of the growth template, a triangular shape. The hexagonal geometry of the dot self-limited profile was in part unexpected: although it is consistent with results previously obtained via AFM analysis of the GaAs self-limited profile on smaller recesses [50], it should be underlined that the physics of growth of the two systems is different. In the latter case, the size of the inverted recess was much smaller than the adatom diffusion length (~ 1 µm), while the tetrahedral holes used for the growth of PQDs in this work have dimensions of several microns, and a similar outcome was not necessarily anticipated.

Figure 6d and g show the chemical composition from the plan view sample. Interestingly, the Aluminium signal is lowest at the first grown layers (at the areas where the Indium is at its highest), similar to what was observed in the cross-sectional samples. Moreover, the Indium elemental map (Fig. 6g) shows a higher concentration at the centre of the sample, where the QD is located, measuring ~ 50 nm in diameter. The surrounding Indium signal comes from the QWs and QWRs where it is observed that the Indium concentration is always much lower than in the QD area. We should note that the detected Indium concentration of the QD in the PV sample is ~ 4%, which is well below the nominal one of 25%. However, the Indium concentration from the cross-sectional sample indicates that the concentration greatly decreases towards the tip of the pyramid. For that particular QD (QD no. 4 out of 9 stacked QDs), the concentration in the cross-sectional sample is slightly higher than the PV sample. This discrepancy is likely to be an artefact of the measurement which is due to averaging effects: the PV sample thickness (80 nm), and the QD naturally being very thin in comparison to the GaAs layers positioned above and below, a strong averaging effect along the electron beam path or a slight tilt in the PV sample would result in a lower Indium atomic fraction estimation. An additional plan view data set can be seen in Fig. S7.

Another striking feature that can be observed from plan-view analysis, which is not observed in the cross-section analysis, is the “Mexican hat “concentration profile in the QD area, with the dot centre richer in Indium when compared to neighbouring areas (Fig. 6d and g). This is also a major finding for electronic structure calculations, as such a specific Indium segregation might influence the resulting underlying symmetry, the excitonic properties, and possibly the effective light /heavy hole mixing for excited states [51].

## Conclusions

This work presents a comprehensive characterisation of InGaAs PQDs. We provide, for the first time to the authors’ knowledge, an insight into the internal structure and the elemental composition of PQDs at the nanoscale thanks to a combination of cross-sectional and plan view imaging. The challenges of the 3D nature of the structure are discussed, and we highlight the effect that slight variations in the sample’s cut have in the final data. This is, there is a strong dependence between the resulting imaging (and geometry shown) and chemical quantification, and caution should be exercised when interpreting the data.

Important findings here reported include the chemical distribution at the facets and QD area. Clear Indium diffusion can be observed at the QD area for all samples, and all QDs, irrespective of the cut. On the other hand, the pyramid sides also exhibit a concentration gradient, and the concentration of Aluminium and Indium differs drastically for the QWR and the QW. The Indium concentration is at its highest on the QWR side and in contrast, the Aluminium concentration is at its lowest, and vice versa at the QW. For stacked QD structures, the QDs were found to be closer together at the apex of the pyramid (first layers grown) while the Indium concentration was seen to progressively increase with growth time. This is rationalised as a direct consequence of the constant nominal growth parameters.

Additionally, the significance of identifying the shape of the self-limited profile, and thus that of the dot, cannot be understated: the dot is hexagonal and not triangular as previously assumed. The exact shape and symmetry of the dot are of great importance, as it is one of the main features that determine the electronic states, and specifically the magnitude of the so-called excitonic fine-structure splitting. Furthermore, the use of AlAs as markers exhibited a flattening, and near absence of Aluminium, at the bottom of the growth profile. This finding is of extreme interest for engineering PQD structures. For example, as an alternative route to selective carrier injection to the one exploited by Chung et al. [20]. And, also of particular interest for the engineering of GaAs/AlGaAs structures, and especially GaAs/AlAs PQDs.

These results provide an important step forward in our understanding of the morphological evolution of MOVPE-grown PQDs, which should lead to the establishment of a better correlation between optical properties and theoretical models. This understanding is critical not only for a more comprehensive electronic state description but also for testing advanced quantum technology schemes, for example the predictions of novel entangled photon emission protocols based on “quantum dumbbells” [33] and/or light-hole/heavy-hole mixing effects exploitation in resonant pumping schemes for cluster state generation [52].

## Supplementary Information

Below is the link to the electronic supplementary material.

Supplementary file1 (DOCX 4471 kb)

## Footnotes

1 Note to the reader: The intuitive rationale for the difference in lateral size of the base is relatively simple; a fast-diffusing atom will try to fill the recess more than a less diffusive species. On the other hand, while the volume grown onto the recess base is different in a specific unit time, the vertical growth rate is fixed by continuity criteria along all the recess surfaces: the bottom layer has to grow in height as much as the lateral sidewalls. The only way these two conditions are fulfilled, is to have a different base for the two atomic systems.
