# Supplementary material for: Towards 3D characterisation of site-controlled InGaAs pyramidal QDs at the nanoscale
Source: J Mater Sci. 2022 Aug 30;57(34):16383–96. doi: 10.1007/s10853-022-07654-2 (PMC9463298; doi:10.1007/s10853-022-07654-2)
Supplement: Supplementary file 1 — Supplementary file1 (DOCX 4471 kb) [file 10853_2022_7654_MOESM1_ESM.docx]

**Supplementary information for**

**Towards 3D characterisation of site-controlled InGaAs pyramidal QDs at the nanoscale**

Kristina M. Holsgrove^1^, Tamsin O’Reilly^1,6^, Simone Varo^2^, Agnieszka Gocalinska**^2^**, Gediminas Juska**^2^**, Demie M. Kepaptsoglou^4,5^, Emanuele Pelucchi^2^ and Miryam Arredondo^1^

**^1^** School of Mathematics and Physics, Queen's University Belfast, UK

**^2^** Tyndall National Institute, “Lee Maltings”, University College Cork, Cork, Ireland

**^3^** Department of Physics, University of York, York YO10 5DD, United Kingdom

^4^ SuperSTEM Laboratory, SciTech Daresbury Campus, Daresbury WA4 4AD, United Kingdom

^5^Department of Physics, University of York, York YO10 5DD, United Kingdom

^6^ University of Glasgow, Glasgow G12 8QQ, United Kingdom

- **Sample growth**


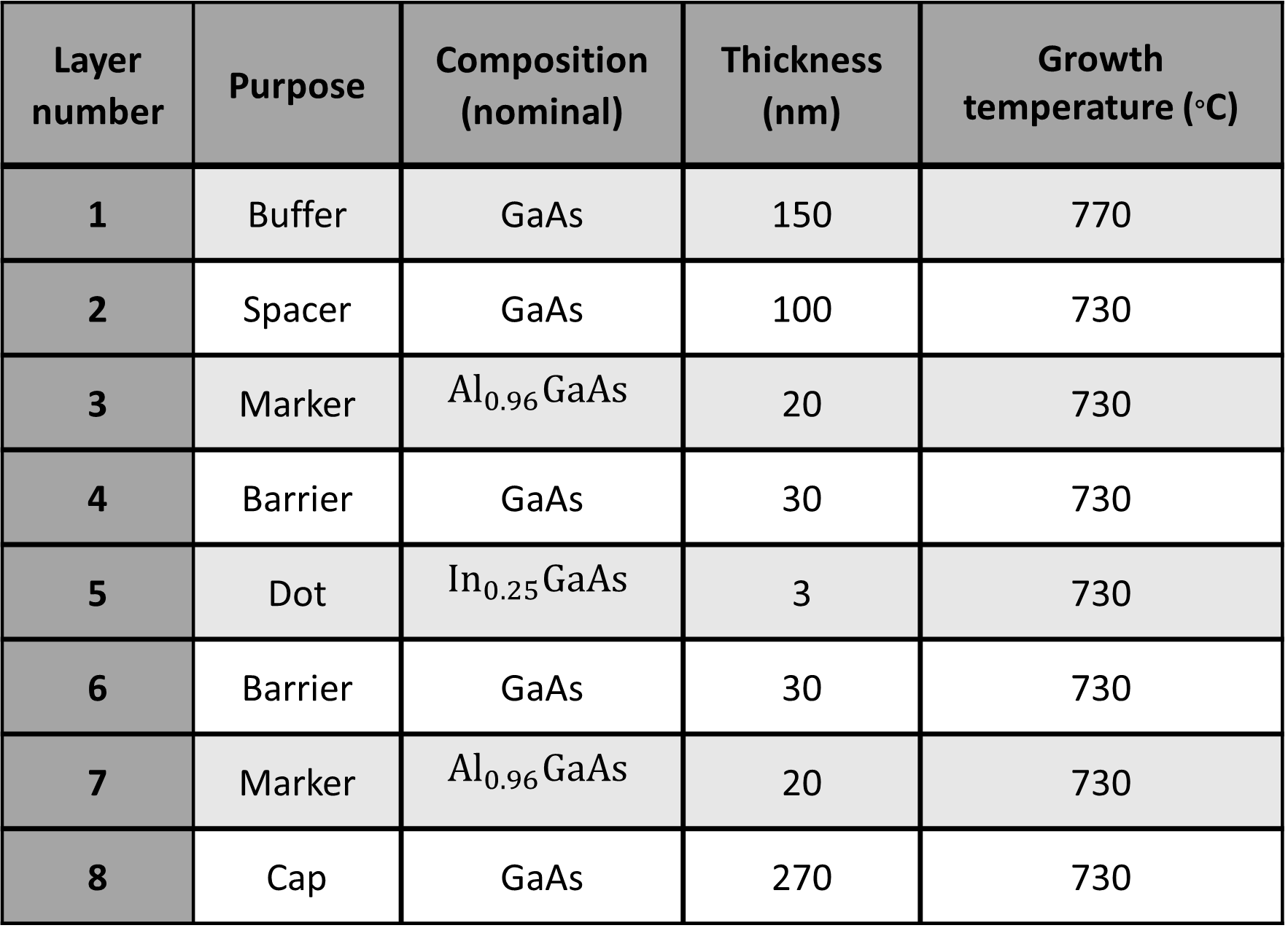


**Table S1.** Growth details for the single QD PQD structure, in the form of pillars


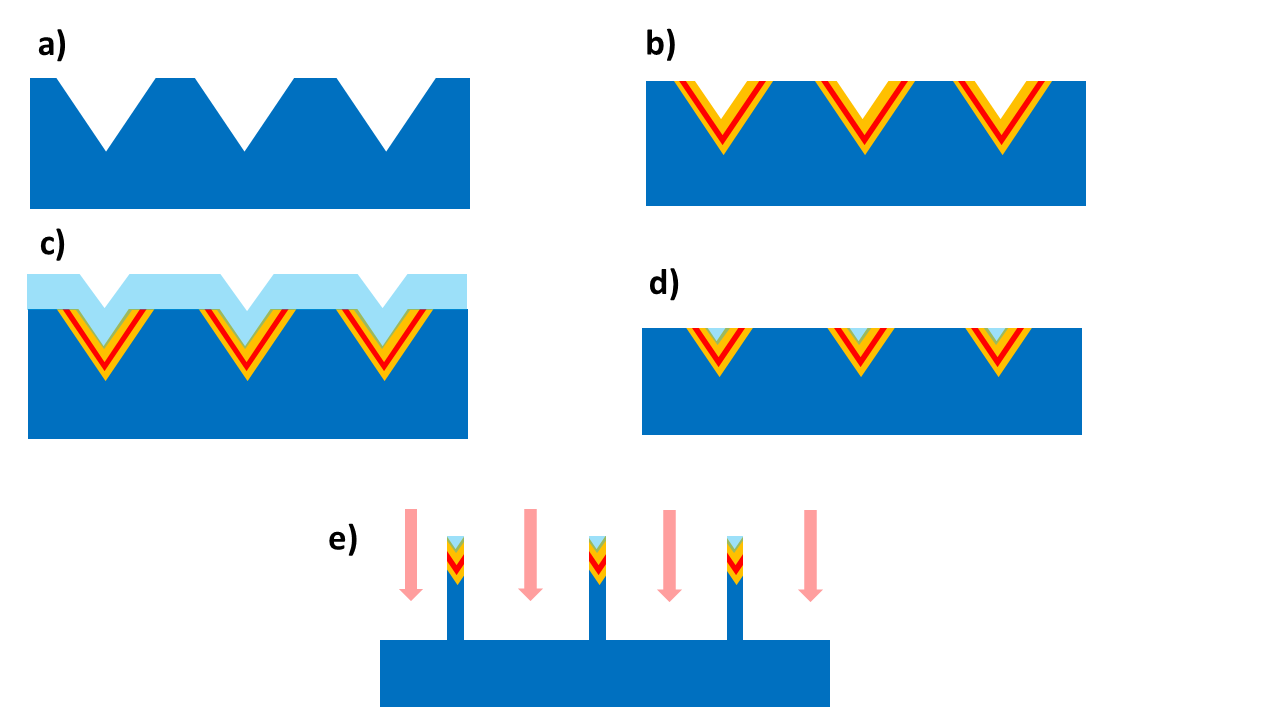


**Figure S1.** Overview sample preparation steps for the nanopillar PDQ structure. A) lithographic patterning, b) MOVPE growth, c) SiO2 deposition, d) planarization and e) dry etching


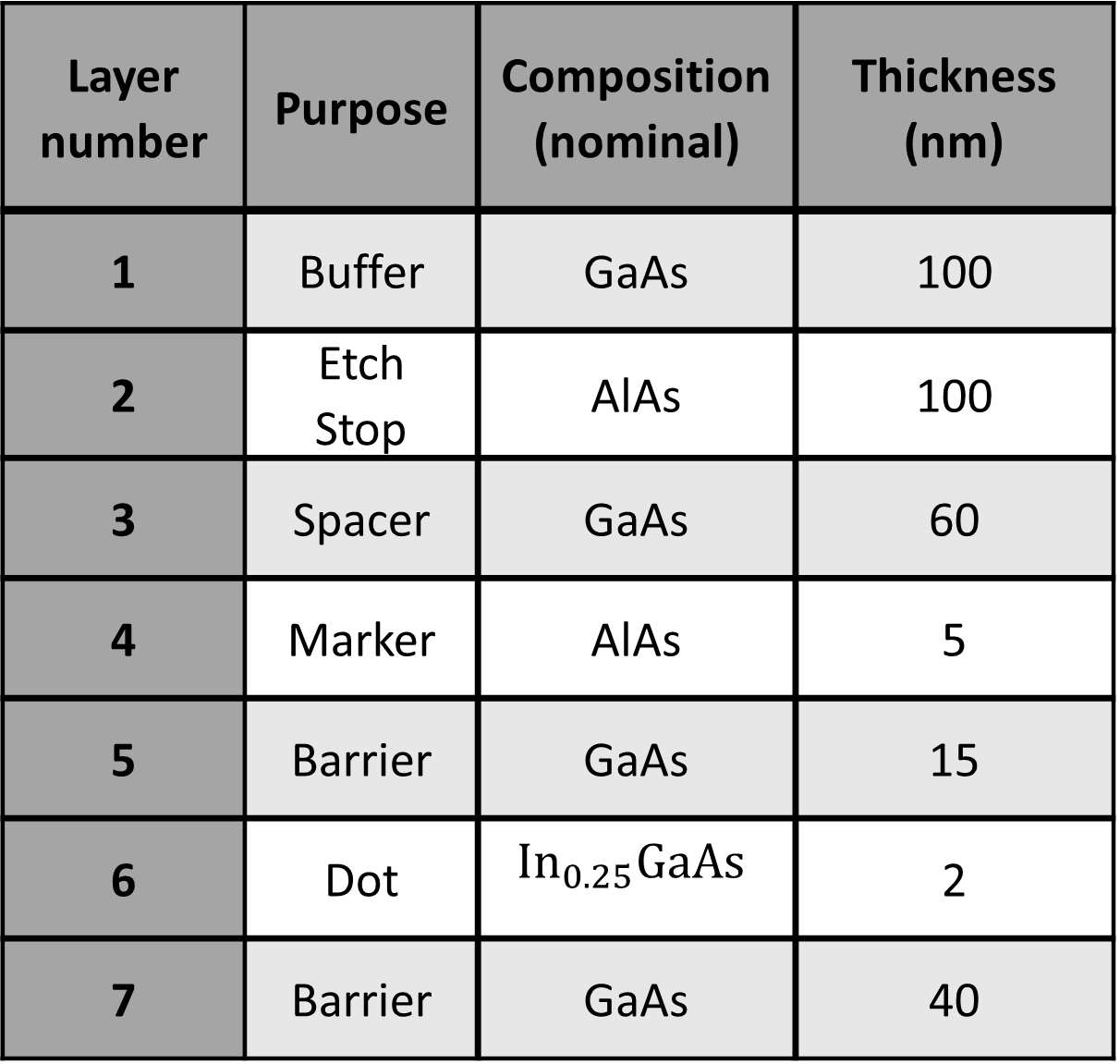


**Table S2.** Growth details for the stacked QDs PQD structure. Layers 4 – 7 are repeated 9 times.

- **Sample preparation**

A Lyra3 TESCAN dual beam scanning electron microscope was used to acquire secondary electron images, and FIB was used to prepare cross-sectional and plan view (PV) TEM samples which were further milled using an Ar^+^ beam in a PIPS II. For the PV samples, e-beam and ion-beam Pt was sputtered on the area of interest, followed by trench milling (1 nA), left cut (500 pA) and undercut (300 pA). The sample was lifted out in situ using the in-built nanomanipulator system and then rotated through 90° using a second needle attached to the stage, a series of attach and detach steps and two stage rotations of 45°. Following this, the sample was attached to a Cu grid, and ion-beam Pt was sputtered (~2.5 um, 100 pA) to protect the new top surface. The sample was then thinned on the grid until the pyramid was exposed, before a final 5 kV polish took the sample <100 nm.


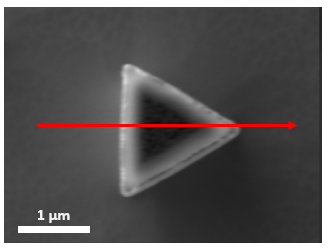


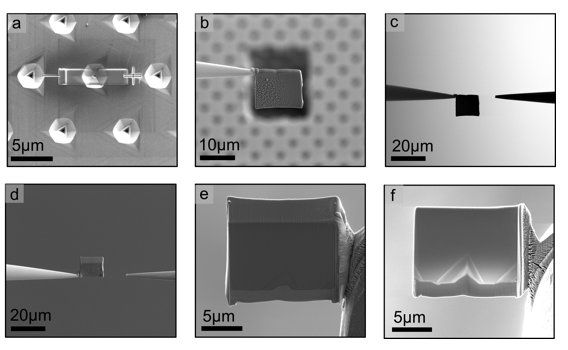


**Figure S2.**  SEM images showing the position and overall process of FIB sample preparation for a cross section pyramidal QD lamellae**.**


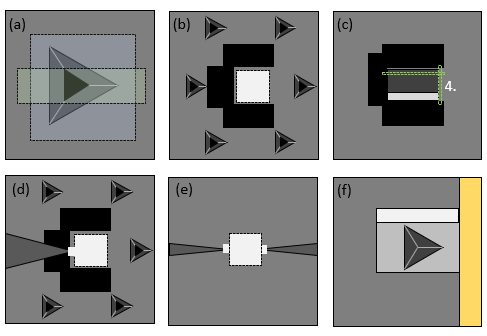


**Figure S3**. Schematic representation of plan-view sample preparation for PQDs.

1. View of bulk sample, deposit e-beam and ion-beam Pt over entire area.
2. Trench mill and left-cut
3. Rotate stage to 0° and undercut.
4. Typical in situ lift out using an needle.
5. Rotate sample 90° using the needle and one attached to the stage (in a sequence of attach/detach, weld/un-weld combined with stage rotation).
6. Once rotated, attach to Cu grid and thin to electron transparency (~100 nm).

- **Strain Analysis**


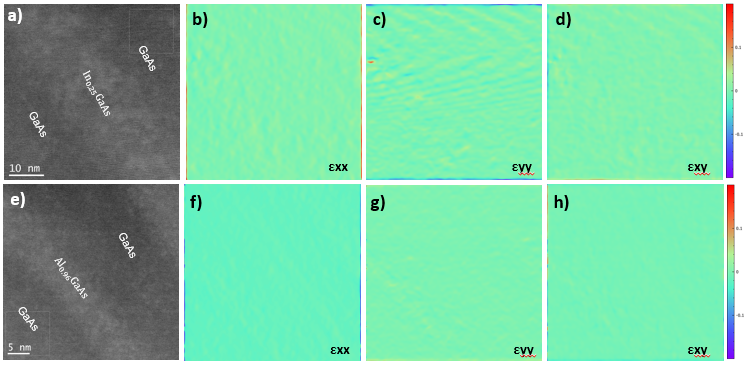


**Figure S4.** Geometrical Phase analysis for the QWR side. a) STEM HAADF image for the GaAs – InGaAs interface, b) – d) corresponding strain maps using the top GaAs area as reference, and e) STEM HAADF image for the GaAs – AlGaAs interface, f) – g) are the corresponding strain maps for e) taking the bottom GaAs as reference area. AlGaAs exhibits a small difference in lattice parameter from GaAs, and substantial strain effects are not to be expected, nor were detected.

- **Additional Chemical Analysis: EDX**


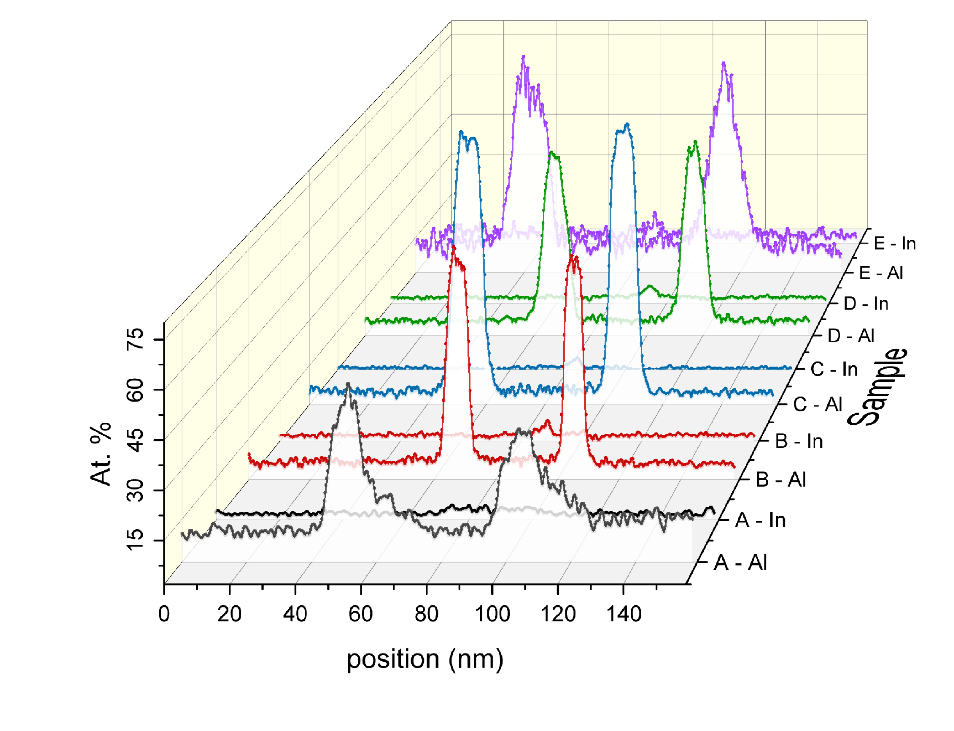


**Figure S5.** Complementary data to Fig. 4 in the main manuscript. Atomic fractions profiles for 5 different cross-sectional samples, A to E, of single PQDs from the same bulk sample, extracted from the representative positions marked in Fig. 3d by the white dotted lines a) Indium content near the pyramid tip and b) Indium and Aluminium content at the quantum well (QW) side. The difference in result are attributed to the slight different alignment of each TEM sample with respect to the PQD apex.

**
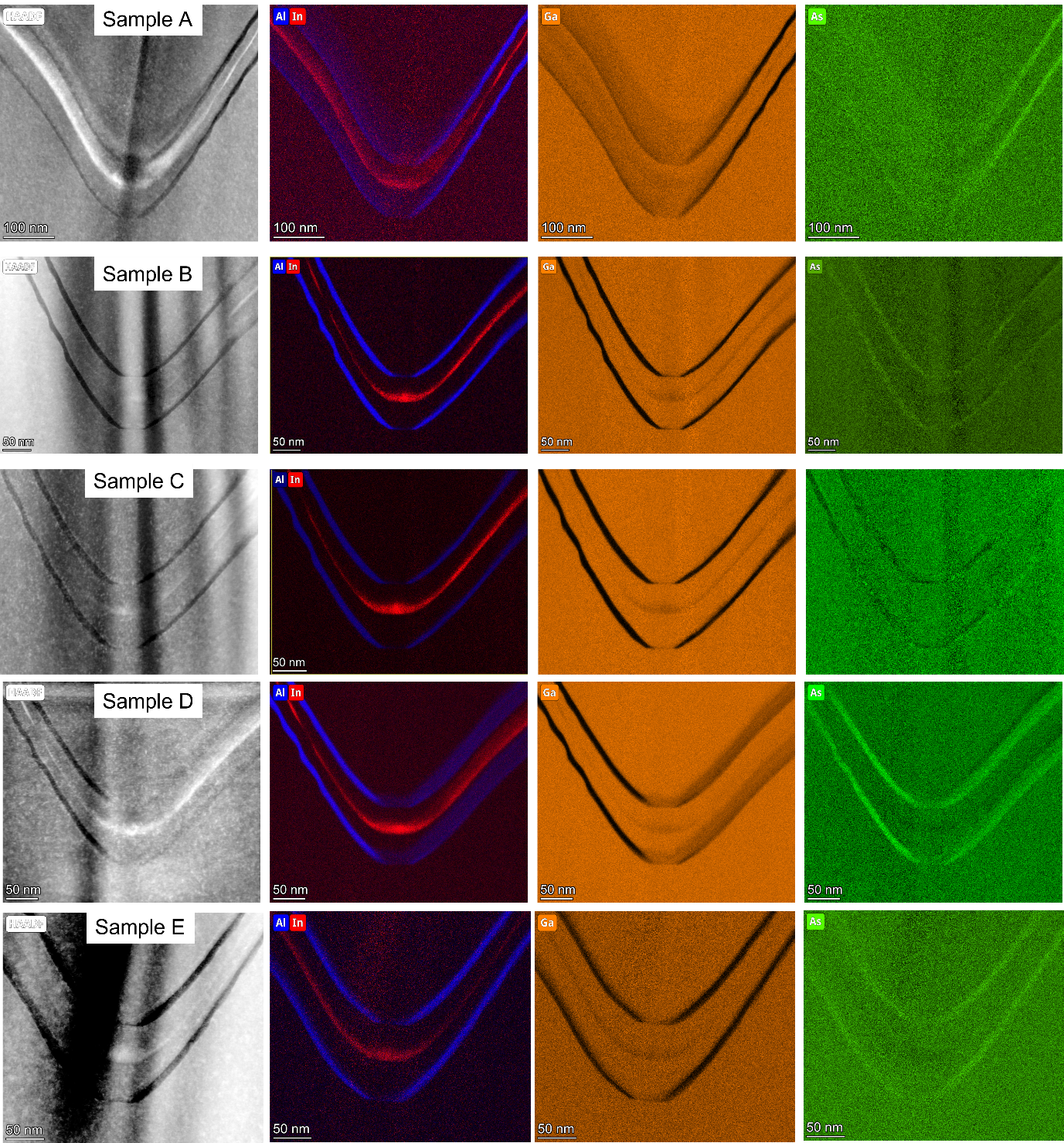
**

**Figure S6.** EDX elemental maps for the samples used for the plots shown in Figs. 4 and S5. The 5 different cross-sectional samples, A to E, are from single PQDs prepared from the same bulk sample


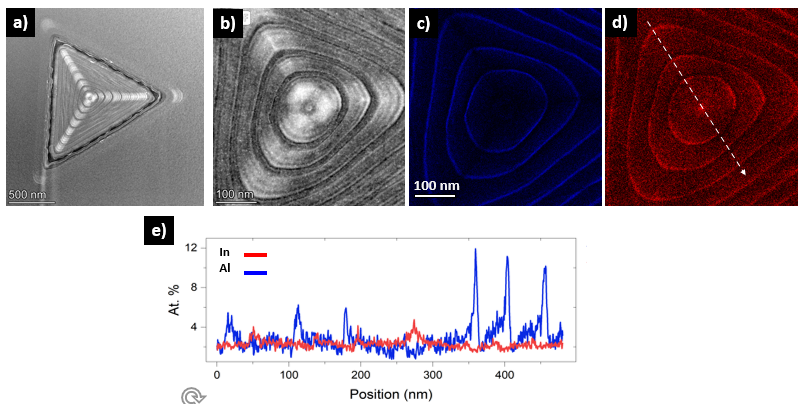


**Figure S7.** Additional plan view data set from the stacked QDs PQD structure shown in Figure 6. a) and b) HAADF STEM plan view overviews roughly acquired from QD4 as marked by the arrow in Fig. 6a), c) and) are the corresponding Al and In EDX elemental maps and e) Al and In at. % line profiles across the centre of the plan view, as marked by the white dotted arrow in d).
